# CNS infections in Greenland: A nationwide register-based cohort study

**DOI:** 10.1371/journal.pone.0171094

**Published:** 2017-02-03

**Authors:** Anne Christine Nordholm, Bolette Søborg, Mikael Andersson, Steen Hoffmann, Peter Skinhøj, Anders Koch

**Affiliations:** 1 Department of Epidemiology Research, Statens Serum Institut, Copenhagen, Denmark; 2 Department of Microbiology and Infection Control, Statens Serum Institut, Copenhagen, Denmark; 3 Department of Infectious Diseases, Rigshospitalet, Copenhagen, Denmark; Washington University, UNITED STATES

## Abstract

**Background:**

Indigenous Arctic people suffer from high rates of infectious diseases. However, the burden of central nervous system (CNS) infections is poorly documented. This study aimed to estimate incidence rates and mortality of CNS infections among Inuits and non-Inuits in Greenland and in Denmark.

**Methods:**

We conducted a nationwide cohort study using the populations of Greenland and Denmark 1990–2012. Information on CNS infection hospitalizations and pathogens was retrieved from national registries and laboratories. Incidence rates were estimated as cases per 100,000 person-years. Incidence rate ratios were calculated using log-linear Poisson-regression. Mortality was estimated using Kaplan-Meier curves and Log Rank test.

**Results:**

The incidence rate of CNS infections was twice as high in Greenland (35.6 per 100,000 person years) as in Denmark (17.7 per 100,000 person years), but equally high among Inuits in Greenland and Denmark (38.2 and 35.4, respectively). Mortality from CNS infections was 2 fold higher among Inuits (10.5%) than among non-Inuits (4.8%) with a fivefold higher case fatality rate in Inuit toddlers.

**Conclusion:**

Overall, Inuits living in Greenland and Denmark suffer from twice the rate of CNS infections compared with non-Inuits, and Inuit toddlers carried the highest risk of mortality. Further studies regarding risk factors such as genetic susceptibility, life style and socioeconomic factors are warranted.

## Introduction

Infections of the central nervous system (CNS) include infection of the meninges and the brain. CNS infections are often severe with a heavy disease burden especially among infants [1].

Inuits of Greenland suffer from high rates of infectious diseases caused by pathogens known to cause CNS infections, including *Streptococcus pneumoniae* and *Mycobacterium tuberculosis* [2, 3]. However, little is known about the full spectrum of CNS infections in the Greenlandic population [4–6]. One study from Canada estimated the nasopharyngeal carriage rates of *Neisseria meningitidis* to be 32.4% among Inuits and only 6% among non-Inuits [7] providing a greater risk of CNS infection to Inuits. Another Canadian study found 1.5 times higher incidence rates (IR) of meningitis among Inuits compared to non-Inuits [4]. One recent study found declining rates of bacterial meningitis in the Arctic but still unreasonably high incidence rates among indigenous arctic people compared to other residents of the North American Arctic [2].

Greenland is part of the kingdom of Denmark. Ninety percent of the Greenlandic population are Inuit, while the majority of the population in Denmark is Caucasian. Life expectancy is approximately 10 years lower in Greenland than in Denmark [8, 9], a difference resembling that observed in aboriginal populations in Alaska and Arctic Canada compared with the Caucasian populations in the USA and Canada.

Although mortality from infectious diseases has declined in both Greenland [10] and Denmark since the 1950s [11], infectious diseases remain a disproportionately severe public health issue in Greenland [12]. As examples, the incidence of invasive pneumococcal disease is approximately seven times higher in Greenland than in Denmark [13, 14] and in Greenland the incidence of tuberculosis is high, approximately 150 per 100,000 [15], while Denmark has had a low incidence for decades [3].

CNS infections are potentially preventable and treatable. Epidemiological knowledge is a powerful tool to ensure proper diagnostics and treatment of CNS infections. We therefore conducted a nationwide study in Greenland with the overall aim to describe the incidence of CNS infections, microbial causes, and short-term mortality in persons (Inuits and non-Inuits) living in Greenland from 1990 to 2012. As we hypothesized that Inuits in Greenland have a higher incidence of CNS infections than non-Inuits, and that this higher incidence is retained in Inuits living in Denmark, we estimated the same figures for Inuits and non-Inuits living in Denmark. Most non-Inuits in Greenland and Denmark are Caucasian Danes. Such increased incidence in Inuits compared with non-Inuits despite the country of residency would indicate a genetic component in the risk of CNS infections in Greenlandic Inuits.

We used national registries and microbiological databases from both countries with the advantage of the health care systems in Greenland and Denmark being comparable in regard to registries and medical guidelines, allowing a direct comparison of incidence and mortality.

## Methods

### Setting

By January 1^st^, 2014, the population of Greenland was 56,282 inhabitants (9), with almost 90% Inuits [16]. The population of Denmark is approximately 100 times larger with 90% Caucasian Danes [17]. About 14,000 Greenlandic Inuits live on a more or less permanent basis in Denmark [18].

In Greenland, more than 82% of the total surface is covered by an ice cap and the sparse population lives in a number of smaller towns and settlements with approximately one fourth of the population living in the capital, Nuuk [9]. The microbiological laboratory in Greenland is situated at Queen Ingrids Hospital (QIH) in Nuuk. This hospital acts as local hospital for Nuuk and as national referral hospital for the rest of Greenland [13]. The laboratory receives microbiological specimens from all Greenlandic hospitals, while specialized hospitals and laboratories in Denmark form a tertiary health system for Greenland. Serological typing and genotyping of bacteria e.g. pneumococci and meningococci are performed at Statens Serum Institut, Copenhagen, Denmark. In both countries, vaccinations against CNS pathogens such as polio virus, morbilli virus, and *Haemophilus influenzae* are part of the routine childhood vaccination program, and more recently *S*. *pneumoniae* was included in the program (in 2007 in Denmark and in 2010 in Greenland). In addition, since 1955, all newborns in Greenland are offered vaccination against *M*. *tuberculosis* (except from a temporary discontinuation 1991–1996 [19]). In Denmark tuberculosis vaccination ceased in the 1980s because of low incidence of the disease [20].

### Study population and definitions

The study was conducted as a nationwide register-based cohort study including all individuals living in Greenland or Denmark at any point between 1990 and 2012 as study population. To be categorized as Inuit at least one parent should be registered as born in Greenland. Based on own place of living (Greenland/Denmark) at time of diagnosis, four groups of persons were defined; Inuits living in Greenland, Inuits living in Denmark, non-Inuits living in Greenland, and non-Inuits living in Denmark. Non-Inuits in Greenland and Denmark are mainly Danes. Follow-up was from 1^st^ of January 1990 or birth, whichever occurred last, until death, emigration or 31^st^ of December 2012, whichever occurred first.

A CNS infection case was defined as a case of meningitis, encephalitis, cerebral abscess, CNS tuberculosis or other. If a CNS infection was coded with tuberculosis (e.g. ‘TB in CNS’ without specification, ‘TB meningitis’ or similar), the case was listed separately as ‘Tuberculosis’ in tables. For a detailed description of the case definitions, please refer to S1 Table. A case was considered laboratory-confirmed if a pathogen had been demonstrated by culture or PCR from the cerebrospinal fluid (CSF), or in case of neurosyphilis by detection of antibodies in CSF or blood, or if a CNS-compatible pathogen was isolated from the blood and the patient was diagnosed with CNS infection.

### Data sources

The populations of Greenland and Denmark were identified through the Civil Registration System (CRS). At birth, every citizen in Greenland and Denmark is assigned a unique personal identification number (CRS number), which identifies the person in all official registries across the two countries [21, 22]. The CRS contains updated information of vital status including sex, time and place of birth, past and present places of living, and ethnicity as defined by parents’ places of birth. This registry was used to define our cohort and to categorize the study population according to their ethnicity and the country they lived in at time of diagnosis.

The National Greenlandic and Danish Inpatient Registries include information on hospitalizations in Greenland since 1987 and in Denmark since 1977 on a person-identifiable basis [23, 24]. From these registries we extracted information of hospital and department, dates of admission and discharge diagnosis codes according to the International Classification of Diseases (ICD) 8^th^ revision until December 1993 and 10^th^ revision thereafter.

Causes of death for persons with prior CNS infection were obtained from the Danish Causes of Death Register, which has included information of causes of deaths in Greenland since 1983 [25].

Information on pathogens in patients with CNS infections was obtained from all available sources, namely from the microbiological laboratory at QIH and from the Department of Microbiology and Infection Control at Statens Serum Institut that serves as microbiological reference laboratory for Greenland. The latter holds information on diagnostic tests and microbiological findings in blood or CSF from hospitals in Greenland since 2000 and from Denmark since 2010. Before 2010, microbiological findings were not recorded on a national level in Denmark except in case of notifiable diseases such as invasive meningococcal disease infection and bacterial meningitis.

### Statistical analyses

Incidence rates (IR) of CNS infections were calculated as the number of events per 100,000 person years (PYRS) with 95% confidence interval (CI). Median age at diagnosis was described with 25–75% interquartile range (IQR). Patients were censored one year after a CNS infection diagnosis. If the interval between diagnoses was more than one year both diagnoses counted. Incidence rate ratios (IRR) were calculated using log-linear Poisson-regression models. All IR and IRR models were estimated using the SAS Genmod procedure. Kaplan-Meier curves were used to illustrate 30-day mortality among patients living in Denmark and patients living in Greenland. Differences in 7-day mortality were estimated through Log-Rank tests in SAS Lifetest procedure. All analyses were made in SAS version 9.4.

### Approvals

The Danish Data Protection Agency (Approval No. 200854–0472) and The Commission for Scientific Research in Greenland (Approval No. 2013–086535) approved the study.

## Results

### Baseline characteristics

In Greenland, 448 cases of CNS infections were identified in the period 1990 to 2012; 250 (56%) men and 198 (44%) women. Median age was 16 years [IQR 2–41 years] and 98% of cases were Inuit. In Denmark, 21,786 cases of CNS infections were identified from 1990 to 2012; 11,405 (52%) men and 10,381 (48%) women. Median age was 37 years [IQR 11–61 years] and 0.7% of cases were Inuit (Table 1).

**Table 1:** Incidence rate (IR) per 100,000 person years of CNS infections in Greenland and Denmark, 1990–2012.

	Greenland					Denmark					IRR (95% CI)**	P-value
CNS infection	N	IR (95% CI)	Inuits (%)	Female sex (%)	Median age * (IQR)	N	IR (95% CI)	Inuits (%)	Female sex (%)	Median age * (IQR)		
Any	448	35.6 (32.4–39.0)	98	44	15.5 (2–41)	21,786	17.7 (17.5–18.0)	0.7	48	37 (11–61)	2.00 (1.82–2.20)	<0.0001
Meningitis	299	23.7 (21.2–26.6)	98	42	11 (1–39)	13,858	11.3 (11.1–11.5)	0.7	48	31 (6–60)	2.02 (1.80–2.27)	<0.0001
Encephalitis	85	6.75 (5.46–8.35)	97	54	24 (9–49)	5,955	4.84 (4.72–4.97)	0.5	50	43 (23–62)	1.47 (1.18–1.82)	0.001
Abscess	20	1.59 (1.02–2.46)	100	45	22.5 (12.5–33)	1,479	1.20 (1.14–1.27)	0.8	40	51 (34–65)	1.50 (0.96–2.33)	0.09
Tuberculosis	22	1.75 (1.15–2.65)	100	36	18.5 (6–41)	261	0.21 (0.19–0.24)	1.5	52	37 (18–55)	7.96 (5.14–12.3)	<0.0001
Other	22	1.75 (1.15–2.65)	96	46	37.5 (4–48)	233	0.19 (0.17–0.22)	2.2	32	46 (31–61)	10.0 (6.45–15.6)	<0.0001

Abbreviations: N: number of cases, IR: incidence rate, IRR: incidence rate ratio, CI: confidence interval, IQR: inter quartile range.

* Median age in years with IQR = IQR1 –IQR3.

** Incidence rate ratio of Greenland compared with Denmark. Adjusted for sex, age group and year.

### Incidence rates

**Fig 1:**
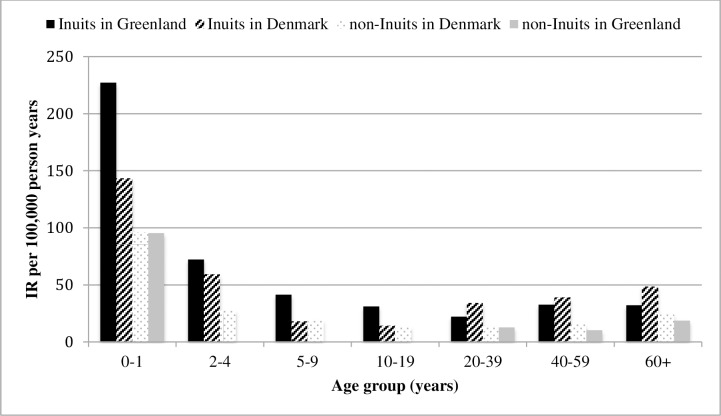
Incidence rates (IR) of CNS infections by age group, ethnicity and country in Greenland and Denmark, 1990–2012. IR peaked among infants and was highest among Inuits compared to non-Inuits in the two countries.

The overall IR of CNS infections in Greenland was 35.6 (95% CI 32.4–39.0) per 100,000 PYRS in the study period 1990–2012. Most of the identified infections were meningitis (IR 23.7 (95% CI 21.2–26.6) per 100,000 PYRS) with a more than two-fold higher IR in Greenland than in Denmark (Table 1). The median age in Greenland was lower for all types of CNS infections as compared to Denmark (Table 1). The incidence of CNS infections was highest among children under 2 years of age and lowest among persons aged 10–39 years (Table 2). Certain groups carried much higher risk of infection. In the meningitis group, the incidence rate ratio (IRR) among Inuit toddlers in Greenland was 3,24 compared to non-Inuits in Denmark (Table 2). Among patients with CNS tuberculosis, the IRR was 8,83 for Inuits in Greenland compared to non-Inuits in Denmark (Table 2). Generally the age specific incidence peaked among infants with a second peak among young adults followed by a slight increase in persons over 40 years of age (Fig 1). This age-specific pattern was similar for the specific diagnoses. Of the four groups, Inuits in Greenland had the highest rates of CNS infections and non-Inuits in Greenland the lowest (Table 2). There was no significant difference between Inuits in Greenland and Inuits in Denmark, and no significant difference between non-Inuits in Greenland and non-Inuits in Denmark. This pattern was seen in all age groups except that Inuits in Greenland had slightly higher rates than Inuits in Denmark in the younger age groups (0–19 years) and vice versa in persons over 20 years of age (Fig 1). There were significant interactions by age between the different population and infection group comparisons (Poisson regression models, p-values not shown) so that the magnitude of IRR between the groups and infections varied over age groups (Table 2), but overall this did not change the general direction of IRR between groups.

**Table 2:** Incidence rate of CNS infection by age, ethnicity and country in Greenland and Denmark, 1990–2012.

CNS infection	Inuits in Greenland				Inuits in Denmark				non-Inuits in Greenland				non-Inuits in Denmark			
	N	PYRS	IR (95%CI)	IRR(95%CI)*	N	PYRS	IR (95%CI)	IRR(95%CI)*	N	PYRS	IR (95%CI)	IRR(95%CI)*	N	PYRS	IR (95%CI)	P-value
**Any**																
**All**	438	1147697	38.2 (34.8–41.9)	2.14 (1.94–2.35)	148	417874	35.4 (30.1–41.6)	2.02 (1.72–2.38)	10	111647	8.96 (4.82–16.6)	0.55 (0.30–1.03)	21638	122531367	17.7 (17.4–17.9)	<0.0001
0–1	95	41812	227 (186–278)	2.52 (2.06–3.09)	25	17534	143 (96.3–211)	1.58 (1.06–2.34)	2	2098	95.3 (23.8–381)	-	2703	2998588	90.1 (86.8–93.6)	<0.0001
2–4	46	63836	72.1 (54.0–96.2)	2.86 (2.13–3.84)	16	27062	59.1 (36.2–96.5)	2.34 (1.43–3.83)	0	2471	-	-	1132	4484365	25.2 (23.8–26.8)	<0.0001
5–9	44	106499	41.3 (30.7–55.5)	2.27 (1.68–3.07)	8	44601	17.9 (8.97–35.9)	0.98 (0.49–1.97)	0	3135	-	-	1329	7297440	18.2 (17.3–19.2)	<0.0001
10–19	58	193852	29.9 (23.1–38.7)	2.35 (1.81–3.05)	11	86577	12.7 (7.04–22.9)	1.00 (0.55–1.80)	0	4417	-	-	1864	14609884	12.8 (12.2–13.4)	<0.0001
20–39	79	368305	21.4 (17.2–26.7)	1.70 (1.36–2.12)	48	141120	34.0 (25.6–45.1)	2.70 (2.03–3.58)	5	39461	12.7 (5.27–30.4)	-	4300	34028406	12.6 (12.3–13.0)	<0.0001
40–59	85	273272	31.1 (25.1–38.5)	2.28 (1.84–2.83)	32	82321	38.9 (27.5–55.0)	2.92 (2.06–4.14)	2	49338	4.05 (1.01–16.2)	-	4546	33483255	13.6 (13.2–14.0)	<0.0001
60+	31	100120	31.0 (21.8–44.0)	1.37 (0.97–1.96)	8	18660	42.9 (21.4–85.7)	1.95 (0.97–3.90)	1	10727	9.32 (1.31–66.2)	-	5764	25629430	22.5 (21.9–23.1)	0.06
**Meningitis**																
**All**	293	1147697	25.5 (22.8–28.6)	2.15 (1.91–2.41)	96	417874	23.0 (18.8–28.1)	1.93 (1.58–2.37)	6	111647	5.37(2.41–12.0)	0.55 (0.25–1.23)	13762	122531367	11.2 (11.0–11.4)	<0.0001
0–1	80	41812	191 (154–238)	2.46 (1.97–3.08)	21	17534	120 (78.1–184)	1.54 (1.00–2.36)	1	2098	47.7 (6.71–338)	-	2329	2998588	77.7 (74.6–80.9)	<0.0001
2–4	36	63836	56.4 (40.7–78.2)	3.24 (2.32–4.53)	10	27062	37.0 (19.9–68.7)	2.11 (1.13–3.94)	0	2471	-	-	781	4484365	17.4 (16.2–18.7)	<0.0001
5–9	27	106499	25.4 (17.4–37.0)	1.90 (1.30–2.79)	6	44601	13.5 (6.04–29.9)	1.00 (0.45–2.24)	0	3135	-	-	974	7297440	13.3 (12.5–14.2)	0.01
10–19	34	193852	17.5 (12.5–24.5)	1.92 (1.37–2.70)	8	86577	9.24 (4.62–18.5)	1.01 (0.50–2.03)	0	4417	-	-	1334	14609884	9.13 (8.65–9.63)	0.003
20–39	43	368305	11.7 (8.66–15.7)	1.55 (1.14–2.09)	27	141120	19.1 (13.1–27.9)	2.54 (1.74–3.70)	4	39461	10.1 (3.80–27.0)	-	2569	34028406	7.55 (7.26–7.85)	<0.0001
40–59	56	273272	20.5 (15.8–26.6)	2.99 (2.30–3.90)	18	82321	21.9 (13.8–34.7)	3.25 (2.05–5.18)	0	49338	-	-	2287	33483255	6.83 (6.56–7.12)	<0.0001
60+	17	100120	17.0 (10.6–27.3)	1.25 (0.77–2.01)	6	18660	32.2 (14.4–71.6)	2.37 (1.06–5.28)	1	10727	9.32 (1.31–66.2)	-	3488	25629430	13.6 (13.2–14.1)	0.12
**Encephalitis**																
**All**	82	1147697	7.14 (5.75–8.87)	1.56 (1.25–1.94)	31	417874	7.42 (5.22–10.5)	1.69 (1.19–2.41)	3	111647	2.69 (0.87–8.33)	0.57 (0.19–1.78)	5924	122531367	4.83 (4.71–4.96)	<0.0001
0–1	10	41812	23.9 (12.9–44.5)	2.21 (1.18–4.16)	2	17534	11.4 (2.85–45.6)	1.05 (0.26–4.23)	1	2098	47.7 (6.71–338)	-	324	2998588	10.8 (9.69–12.0)	0.09
2–4	3	63836	4.70 (1.52–14.6)	0.69 (0.22–2.15)	6	27062	22.2 (9.96–49.4)	3.25 (1.45–7.28)	0	2471	-	-	306	4484365	6.82 (6.10–7.63)	0.04
5–9	9	106499	8.45 (4.40–16.2)	2.03 (1.05–3.94)	2	44601	4.48 (1.12–17.9)	1.08 (0.27–4.32)	0	3135	-	-	304	7297440	4.17 (3.72–4.66)	0.17
10–19	17	193852	8.77 (5.45–14.1)	3.12 (1.92–5.08)	3	86577	3.47 (1.12–10.7)	1.23 (0.40–3.84)	0	4417	-	-	410	14609884	2.81 (2.55–3.09)	<0.001
20–39	18	368305	4.89 (3.08–7.76)	1.28 (0.80–2.04)	13	141120	9.21 (5.35–15.9)	2.41 (1.40–4.17)	1	39461	2.53 (0.36–18.0)	-	1298	34028406	3.81 (3.61–4.03)	0.01
40–59	15	273272	5.49 (3.31–9.10)	1.14 (0.68–1.89)	5	82321	6.07 (2.53–14.6)	1.25 (0.52–3.00)	1	49338	2.03 (0.29–14.4)	-	1620	33483255	4.84 (4.61–5.08)	0.80
60+	10	100120	9.99 (5.37–18.6)	1.54 (0.83–2.86)	0	18660	-	-	0	10727	-	-	1662	25629430	6.48 (6.18–6.80)	-
**Abscess**																
**All**	20	1147697	1.74 (1.12–2.70)	1.71 (1.10–2.66)	12	417874	2.87 (1.63–5.06)	3.21 (1.81–5.66)	0	111647	-	-	1467	122531367	1.20 (1.14–1.26)	0.0003
0–1	2	41812	4.78 (1.20–19.1)	4.34 (1.04–18.1)	2	17534	11.4 (2.85–45.6)	10.4 (2.50–43.3)	0	2098	-	-	33	2998588	1.10 (0.78–1.55)	0.016
4.78 (1.20–19.1)	4.34 (1.04–18.1)
2–4	1	63836	1.57 (0.22–11.1)	3.06 (0.41–22.6)	0	27062	-	-	0	2471	-	-	23	4484365	0.51 (0.34–0.77)	0.56
5–9	1	106499	0.94 (0.13–6.67)	2.21 (0.30–16.2)	0	44601	-	-	0	3135	-	-	31	7297440	0.42 (0.30–0.60)	0.65
10–19	3	193852	1.55 (0.50–4.80)	2.79 (0.88–8.84)	0	86577	-	-	0	4417	-	-	81	14609884	0.55 (0.45–0.69)	0.20
20–39	11	368305	2.99 (1.65–5.39)	3.40 (1.86–6.21)	4	141120	2.83 (1.06–7.55)	3.31 (1.23–8.87)	0	39461	-	-	298	34028406	0.88 (0.78–0.98)	<0.001
40–59	1	273272	0.37 (0.05–2.60)	0.24 (0.03–1.72)	6	82321	7.29 (3.27–16.2)	5.41 (2.42–12.1)	0	49338	-	-	499	33483255	1.49 (1.37–1.63)	0.001
60+	1	100120	1.00 (0.14–7.09)	0.50 (0.07–3.58)	0	18660	-	-	0	10727	-	-	502	25629430	1.96 (1.79–2.14)	0.54
**Tuberculosis**																
**All**	22	1147697	1.92 (1.26–2.91)	8.83 (5.70–13.7)	4	417874	0.96 (0.36–2.55)	4.31 (1.60–11.6)	0	111647	-	-	257	122531367	0.21 (0.19–0.24)	<0.0001
0–1	1	41812	2.39 (0.34–17.0)	7.99 (1.01–63.0)	0	17534	-	-	0	2098	-	-	9	2998588	0.30 (0.16–0.58)	0.30
2–4	2	63836	3.13 (0.78–12.5)	7.79 (1.81–33.6)	0	27062	-	-	0	2471	-	-	18	4484365	0.40 (0.25–0.64)	0.09
5–9	4	106499	3.76 (1.41–10.0)	21.1 (6.87–64.6)	0	44601	-	-	0	3135	-	-	13	7297440	0.18 (0.10–0.31)	<0.001
10–19	4	193852	2.06 (0.77–5.50)	10.4 (3.65–29.5)	0	86577	-	-	0	4417	-	-	29	14609884	0.20 (0.14–0.29)	<0.01
20–39	5	368305	1.36 (0.57–3.26)	6.02 (2.44–14.9)	1	141120	0.71 (0.10–5.03)	3.06 (0.43–22.0)	0	39461	-	-	77	34028406	0.23 (0.18–0.28)	<0.01
40–59	5	273272	1.83 (0.76–4.40)	9.61 (3.87–23.9)	3	82321	3.64 (1.18–11.3)	21.2 (6.62–67.6)	0	49338	-	-	63	33483255	0.19 (0.15–0.24)	<0.0001
60+	1	100120	1.00 (0.14–7.09)	5.29 (0.73–38.3)	0	18660	-	-	0	10727	-	-	48	25629430	0.19 (0.14–0.25)	0.41
**Other**																
**All**	21	1147697	1.83 (1.19–2.81)	11.0 (7.03–17.3)	5	417874	1.20 (0.50–2.87)	9.56 (3.92–23.3)	1	111647	0.90 (0.13–6.36)	4.20 (0.59–30.0)	228	122531367	0.19 (0.16–0.21)	<0.0001
0–1	2	41812	4.78 (1.20–19.1)	17.9 (3.81–84.4)	0	17534	-	-	0	2098	-	-	8	2998588	0.27 (0.13–0.53)	0.02
2–4	4	63836	6.27 (2.35–16.7)	70.4 (17.6–281)	0	27062	-	-	0	2471	-	-	4	4484365	0.09 (0.03–0.24)	<0.0001
5–9	3	106499	2.82 (0.91–8.73)	29.4 (7.59–114)	0	44601	-	-	0	3135	-	-	7	7297440	0.10 (0.05–0.20)	0.001
10–19	0	193852	-	-	0	86577	-	-	0	4417	-	-	10	14609884	0.07 (0.04–0.13)	0.83
20–39	2	368305	0.54 (0.14–2.17)	3.18 (0.78–13.0)	3	141120	2.13 (0.69–6.59)	12.8 (4.00–40.8)	0	39461	-	-	58	34028406	0.17 (0.13–0.22)	0.003
40–59	8	273272	2.93 (1.46–5.85)	12.4 (6.00–25.7)	0	82321			1	49338	2.03 (0.29–14.4)	-	77	33483255	0.23 (0.18–0.29)	<0.0001
60+	2	100120	2.00 (0.50–7.99)	7.85 (1.92–32.1)	2	18660	10.7 (2.68–42.9)	52.4 (12.8–215)	0	10727	-	-	64	25629430	0.25 (0.20–0.32)	0.0003

Abbreviations: N: number of cases, PYRS: person years, CI: confidence interval IR: incidence rate, IRR: Incidence rate ratios.

* Incidence rate ratios compared with non-Inuits in Denmark. Adjusted for sex.

In Greenland, the IR of clinically diagnosed CNS infections was significantly higher at coastal hospitals compared with Nuuk (IR coastal hospital 39.5 95% CI (35.7–43.7) vs. IR Nuuk 23.7 95% CI (18.9–29.7). IRR 1.56 95% CI (1.21–2.00), p = 0.0003), whereas the IR of laboratory-confirmed CNS infections was lower at coastal hospitals though this difference was not significant (IR coastal hospitals 6.97 95% CI (5.48–8.87) vs. IR Nuuk 9.28 95% CI (6.45–13.4). IRR 0.67 95% CI (0.43–1.04), p = 0.07).

### Microbiology

Microbiologically verified CNS-infections accounted for 21% of CNS infection cases (95 patients) in Greenland 1990–2012, hereof 82 with bacterial meningitis (Table 3), nine with viral meningitis, one with viral encephalitis, and three with neurosyphilis.

**Table 3:** Incidence rate of laboratory-confirmed bacterial meningitis and the distribution of bacterial agents in meningitis cases in Greenland 1990–2012 and in Denmark 2011–2012 identified by culture in either cerebrospinal fluid (CSF) or blood.

Bacterial agents	Greenland 1990–2012		Denmark 2011–2012	
	IR 6.27 95% CI (5.03–7.82) CSF or blood (N = 82)		IR 4.58 95% CI (4.20–4.99) CSF or blood (N = 308)	
	N	%	N	%
*S*. *pneumoniae*	55	67	118	38
*N*. *meningitidis*	12	15	24	8
*H*. *influenzae*	5	6.1	15	5
*Staphylococcus aureus*	4	4.9	21	7
*Escherichia coli*	1	1.2	19	6
*Listeria monocytogenes*	1	1.2	20	6
*Streptococcus* spp.	2	2.4	35	11
*M*. *tuberculosis*	2	2.4	0	0
Other	0	0	56	18

In Denmark 2011–12, 26% of CNS infections were microbiologically verified (509) of which 308 were bacterial meningitis (Table 3). In both countries *S*. *pneumoniae* was the most frequent pathogen, found significantly more often in meningitis cases in Greenland (in 67% and 38% of cases in Greenland and Denmark, respectively). *Neisseria meningitidis* was relatively more common in Greenland compared with Denmark.

### Mortality

**Fig 2:**
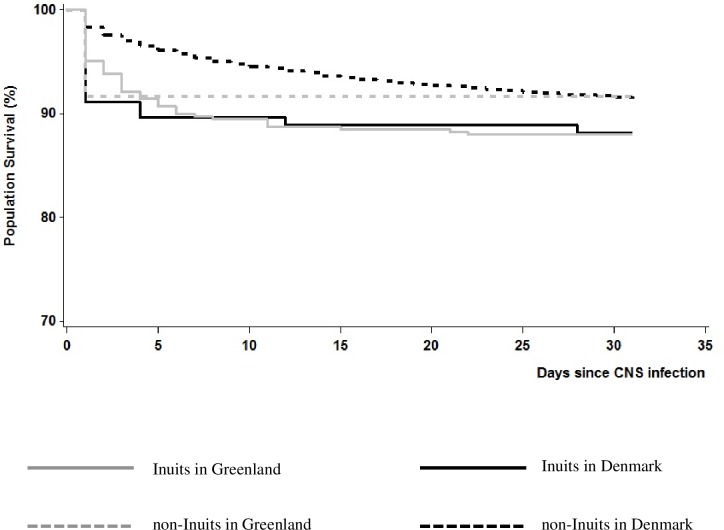
One-month mortality after CNS infection among Inuits and non-Inuits in Greenland and Denmark, 1990–2012. The highest mortality was observed the first week after infection. Inuits in Greenland and Denmark carried the highest risk of mortality compared to non-Inuits in the two countries.

One-month mortality was higher among Inuits in Greenland and Denmark compared with non-Inuits in the two countries (Fig 2). Most deaths occurred the first week following infection. Seven-days mortality after CNS infection was two times higher in Inuits in Greenland and in Denmark compared with non-Inuits in Denmark (only one non-Inuit in Greenland died) (Table 4). Inuit children under two years of age in Greenland and in Denmark had a five-fold higher mortality compared with non-Inuit children in Denmark (Table 5). In Greenland, mortality rates from CNS infections in Nuuk and coastal hospitals were of the same magnitude (12.5% (95% CI 4.25–20.04) vs. 10.14% (95% CI 6.95–13.23)).

**Table 4:** Seven days mortality from CNS infections by ethnicity and country in Greenland and Denmark, 1990–2012.

	Inuits in Greenland		Inuits in Denmark		non-Inuits in Greenland		non-Inuits in Denmark		P-value
	CFR	95% CI	CFR	95% CI	CFR	95% CI	CFR	95% CI	
Any	10.5	(7.5–13.4)	10.5	(5.4–15.4)	12.5*	(0–31.5)	4.8	(4.5–5.1)	<0.0001
Meningitis	12.6	(8.7–16.4)	11.2	(4.7–17.3)	25.0	(0–54.0)	6.2	(5.8–6.6)	<0.0001
Encephalitis	4.8	(0–8.6)	7.1	(0–15.9)	-	-	1.6	(1.2–1.9)	0.01
Abscess	5.9	(0–16.1)	20	(0–39.4)	-	-	3.9	(2.8–5.0)	0.02
Tuberculosis	6.3	(0–17.1)	-	-	-	-	3.2	(0.7–5.7)	0.50
Other	10.5	(0–22.7)	-	-	-	-	0	-	-

Abbreviations: CFR: Case fatality rate, CI: confidence interval.

* Only 1 person.

**Table 5:** Seven days mortality from CNS infections by age, ethnicity and country in Greenland and Denmark, 1990–2012.

	Inuits in Greenland		Inuits in Denmark		non-Inuits in Greenland		non-Inuits. Denmark		P-value
Age	CFR	95% CI	CFR	95% CI	CFR	95% CI	CFR	95% CI	
0–1	11.7	(5.1–17.9)	8.3	(0–18.5)	-	-	2.2	(1.6–2.7)	<0.0001
2–4	9.8	(0.4–18.2)	-	-	-	-	1.8	(1.0–2.6)	<0.001
5–9	7.0	(0–14.2)	-	-	-	-	0.7	(0.2–1.1)	<0.0001
10–19	4.4	(0–10)	11.1	(0–28.5)	-	-	1.5	(1.0–2.1)	0.02
20–39	1.6	(0–4.5)	17.8	(7–27.3)	-	-	2.1	(1.7–2.6)	<0.0001
40–59	18.1	(9.7–25.7)	7.7	(0–17.0)	-	-	4.1	(3.5–4.7)	<0.0001
60+	20.0	(4.6–32.9)	12.5	(0–31.5)	100*	-	11.1	(10.2–11.9)	0.0001

Abbreviations: CFR: Case Fatality Rate, CI: Confidence interval.

* Only 1 person.

## Discussion

To our knowledge, this is the first study to describe incidence and mortality from the total spectrum of CNS infections in Greenland. We found that Inuits in both Greenland and Denmark had twice the rate of CNS infection compared with non-Inuits in Greenland and in Denmark, with Inuit children under 2 years of age carrying a particular high risk of morbidity and mortality from these infections.

The higher IR of CNS infections in Greenland compared with Denmark was primarily driven by a high IR of meningitis in Greenland. The IR of encephalitis and cerebral abscesses was similar in Greenland and Denmark (Table 1) and of the same magnitude as that observed in studies from the USA [26, 27].

The difference in IR of CNS infections between Inuits and non-Inuits in Greenland and Denmark may be ascribed to differences in genetic or environmental factors, or a combination. Rates of other infectious diseases including invasive bacterial infections and respiratory tract infections are known to be higher in Inuits and American native populations compared with Danes and Caucasians living in Arctic areas, respectively [28, 29]. Differences in socioeconomic status could explain the higher IR in Inuits with factors such as smoking and household crowding being more prevalent in Inuits [29]. These factors and upper respiratory tract infections increase the risk of invasive meningococcal disease [30] and could contribute to the higher risk of CNS infections in Inuits compared with non-Inuits. However, it was surprising that the IR of CNS infections in Inuits was equally high among Inuits in Greenland and Inuits in Denmark (Table 2). While living conditions for non-Inuits living in Greenland may be of a higher standard than those of Inuits in Greenland, primarily due to better employment and often better paid jobs, the majority of Inuits in Denmark are more likely to live like Danes in Denmark [18]. Therefore, if environmental factors played a greater role in the risk of infection rather than genetic factors, we would expect to find lower IR of CNS infections among Inuits in Denmark than in Greenland. The fact that we found the same incidence of CNS infections in the two groups in spite of different living conditions could indicate that genetic factors could play a role in the susceptibility to CNS infection.

Infants and toddlers had the highest IR of CNS infections, both overall and by specific diagnoses, followed by declining rates in older age groups and slightly increasing IR among the elderly. As most cases of CNS infections were meningitis caused by *S*. *pneumoniae* this finding is not surprising given the well-known U-shaped age-specific pattern of such infections seen in many populations. The burden of disease was heaviest for infants and toddlers in both Greenland and Denmark but for all types of CNS infections we found younger patients in Greenland compared to Denmark (Table 1). This could be due to a higher incidence of CNS pathogens in Greenland or perhaps Inuits in Greenland are more prone to be sick from CNS infections earlier in life. The fact that the IRR among Inuit infants and toddlers in Greenland was 2.46 and 3.24 per 100,000 person years (PYRS) respectively, compared to non-Inuits in Denmark (Table 2), underlines the serious burden of meningitis among children in Greenland. CNS tuberculosis was far more common in Greenland compared to Denmark, which was expected, (IRR 8,83 per 100,000 PYRS), though it was somewhat surprising that children suffered to such a degree from this serious disease (IR 21 per 100,000 PYRS) in Greenland (which must be interpreted with caution due to the low number of children with CNS TB).

Only one of four CNS infections in Greenland was microbiologically verified. This was not surprising given the limited microbiological facilities in Greenland, difficult sample transportation conditions, and lack of equipment to test for e.g. viral agents. The relatively small number of verified CNS infections in Denmark might reflect that the nationwide register to collect data on all results from clinical microbiological departments was established in 2010 in Denmark. Thus, our data may not reflect the true rate of laboratory verified infections. However, there is no reason to believe that there is a bias regarding the distribution of identified pathogens in Denmark as shown in Table 3. The figures in Table 3 underline the importance of *S*. *pneumoniae* in Greenland with a markedly higher proportion of *S*. *pneumoniae* in culture-positive samples in Greenland compared with Denmark. The high burden of invasive disease from this bacterium in Native Arctic populations, including Inuits, has been described previously [13, 28]. There was a reduction in IR of *S*. *pneumoniae* meningitis in the years from 2009 compared with previous years in Greenland. The explanation for this IR decrease may partly be that the 13-valent conjugate *S*. *pneumoniae* vaccine Prevnar13® was introduced in the Greenlandic childhood vaccine program in 2010 (2007 in Denmark). This vaccine has shown to be effective in Alaskan natives [31] and it may therefore be expected that the incidence of *S*. *pneumoniae* meningitis in Greenland will decrease further in the years to come. Likewise, the proportion of cases caused by *N*. *meningitidis* was higher in Greenland than in Denmark which is somewhat surprising as this microorganism is sensitive to long sample transportation time, which often occurs in Greenland, and difficult to culture from CSF, especially if the lumbar puncture is taken after initiation of antibiotics [32]. This suggests that the incidence of *N*. *meningitidis* meningitis in Greenland is most likely underestimated.

A recently published study on bacterial meningitis in the North American Arctic including Greenland, based on notifications to the Healthcare Facilities [2], showed rates of bacterial meningitis that were much lower than those presented in this study. While we estimated an laboratory-confirmed bacterial meningitis incidence rate of 6.27 per 100,000 per year, the North American Arctic study reported an overall incidence rate of 5.0 per 100,000 per year for the period 2000–2010. The main reason for this discrepancy is that only laboratory verified cases (*S*. *pneumoniae*, *N*. *meningitidis* or *H*. *influenzae*) are mandatory notifiable. We estimated a meningitis incidence rate of 23.7 per 100,000 indicating that restricting cases to those with a laboratory verified diagnosis may underestimate the true incidence rates of e.g. meningitis.

The overall incidence of CNS infections in Greenland was almost 50% higher on the coast (outside the capital of Nuuk) compared with Nuuk. In contrast, the IR of laboratory-confirmed CNS infections was highest in Nuuk. This reflects the fact that the microbiological laboratory is placed in Nuuk, and the likelihood of obtaining a laboratory confirmed microbiological diagnosis in case of a CNS infection is therefore substantially higher at QIH as compared with coastal hospitals. Test results may be falsely negative, as specimens need to be transported to Nuuk and bacteria may not survive transportation under changing temperatures [33]. Therefore, physicians at coastal hospitals may be less inclined to take microbiological specimens compared with physicians in Nuuk, and rates based on laboratory confirmed cases in Greenland may be biased towards underreporting from coastal hospitals.

Seven-days mortality was higher in Greenland than in Denmark with Inuits having a higher mortality than non-Inuits regardless of their country of residence. Of particular concern, we found Inuit children in Greenland under two years of age to suffer a more than five-fold increased risk of death as compared with non-Inuit children in Denmark. While differences in treatment facilities in Greenland may be part of the explanation for the higher over-all mortality in Greenland than in Denmark, it does not explain the equally high mortality in Inuits in Denmark. Access to health service is free-of-charge for all citizens of Denmark and although there may be differences in hospital contact patterns, co-morbidity or socio-economic factors that increase mortality in CNS infections, we hypothesize that genetically determined factors increase mortality from CNS infections among Inuits.

The major strengths of this study are its nationwide design and long follow-up time. Every citizen of both Greenland and Denmark is identified through the CRS number that uniquely identifies the person in national registers and allows for precise calculation of person years. Using parents place of birth as registered in the CRS as a proxy for ethnicity allowed us to identify Inuits and non-Inuits in Greenland and Denmark in order to compare incidence and mortality rates. Data registration is compatible in the two countries. We used all available sources on hospital admissions, diagnoses and microbiological tests and were not restricted to e.g. notifiabel cases, which is much less complete.

The limitations in this study include reliance on the registry-based discharge diagnoses. As there is a lack of microbiological facilities in Greenland, relatively more diagnoses of CNS infections must be made without laboratory confirmation. Whether such possible misclassification results in over-diagnosis or under-diagnosis of CNS infections in Greenland is unclear. However, the Greenlandic National Inpatient Register has recently been validated and was shown to be both complete and correct compared with information in medical files [34]. Another limitation is that we may underestimate the incidence of CNS infections in both Greenland and Denmark, as there has been no active effort to detect cases and identify pathogens in this retrospective study [5, 35]. Also, the size of the population of Greenland is limited (N = 56,000) and the group of non-Inuits in Greenland likewise small (approximately 10% of the population). This gives little statistical power to comparisons with this group. We did not have access to medical records, so the effects of potential risk factors such as maternal smoking, number of siblings, crowding, and low birth weight [36] could not be addressed in this study.

In conclusion, the incidence of CNS infection was two-fold higher in Greenland as compared with Denmark. Inuits in Greenland and Denmark had higher and comparable rates of meningitis than non-Inuits in Denmark and Greenland. Inuits carried higher morbidity and mortality rates, Inuit children under 2 years of age being especially vulnerable. Our study underlines important differences in the incidence of CNS infections between Inuits and non-Inuits in Greenland and Denmark. We were not able to address these differences in this study and further research regarding risk factors such as genetic susceptibility and socioeconomic status is of great concern.

## Supporting information

S1 Table Appendix 1. Diagnoses codes categorized by type of CNS infection.
(DOCX)
