# Supplementary material for: CNS infections in Greenland: A nationwide register-based cohort study
Source: PLoS One. 2017 Feb 3;12(2):e0171094. doi: 10.1371/journal.pone.0171094 (PMC5291447; doi:10.1371/journal.pone.0171094)
Supplement: S1 Table — Diagnoses codes categorized by type of CNS infection. (DOCX) [file pone.0171094.s001.docx]

**S1 Table. Appendix 1**

Diagnoses codes categorized by type of CNS infection

| **CNS infection** | **Code*** | **Diagnosis** |
| --- | --- | --- |
| Meningitis  ICD 8*  ICD 10* | 3609  4509  4519  4599  32009  32019  32080  32089  32090  32099  DA321  DA87  DB003  DB010  DB020  DB051  DG00  DG01  DG02  DG03 | Meningitis meningococcia  Meningitis serosa coxsackie  Meningitis serosa echo  Meningitis serosa  Meningitis purulenta per haemophilum influenza  Meningitis purulenta per pneumococcum  Meningitis purulenta per streptococcum  Meningitis per organismos alios specificatos  Meningitis  Meningitis per organismos non specificatos  Listerial meningitis and meningoencephalitis  Viral meningitis (all subgroups)  Herpesviral meningitis  Varicella meningitis  Zoster meningitis  Measles complicated by meningitis  Bacterial meningitis (all subgroups)  Meningitis in bacterial diseases classified elsewhere (all subgroups)  Meningitis in other infectious and parasitic diseases classified elsewhere (all subgroups)  Meningitis due to other and unspecified causes |
| Encephalitis  ICD 8*  ICD 10* | 6209  6219  6299  6309  6329  6399  6499  6599  32091  32300  32303  32308  32309  DA811  DA812  DA83  DA84  DA85  DA86  DB004  DB011  DB020  DB050  DG04  DG05 | Encephalitis japonica  Encephalitis equina occidentalis  Encephalitis virosa per mosquitos alia et non specificata  Meningoencephalitis verno-estivalis russica  Encephalitis Europa centralis  Encephalitis virosa per ixodidam aliam et non specificata  Encephalitis virosa per arthropoda alia  Encephalitis virosa non specificata  Meningoencephalitis  Encephalitis acuta infectiosa  Encephalomyelitits disseminate  Encephalitis, encephalomyelitis et myelitis alia definite  Encephalitis, encephalomyelitis et myelitis  Subacute sclerosing panencephalitis  Progressive multifocal leukoencephalopathy  Mosquito-borne viral encephalitis (all subgroups)  Tick-borne encephalitis (all subgroups)  Other viral encephalitis not elsewhere classified (all subgroups)  Unspecified viral encephalitis (all subgroups)  Herpesviral encephalitis  Varicella encephalitis  Zoster encephalitis  Measles complicated by encephalitis  Encephalitis, myelitis and encephalomyelitis (all subgroups)  Encephalitis, myelitis and encephalomyelitis in diseases classified elsewhere (all subgroups) |
| Abscess  ICD 8*  ICD 10* | 32200  32202  32204  32208  32209  DG06  DG07 | Abscessus cerebri, cerebelli  Abscessus subduralis intracranialis  Abscessus epiduralis intracranialis  Abscessus intracranialis et intraspinalis alius definitus  Abscessus intracranialis et intraspinalis  Abscessus et granuloma intracraniale (all subgroups)  Abscessus intracraniale et intraspinalis in diseases classified elsewhere (all subgroups) |
| CNS tuberculosis  ICD 8*  ICD 10* | 1300  1301  1308  1309  1390  1399  DA17 | Tuberculosis meningum sine affectione systematis nervosa centralis  Tuberculosis meningum cum affectione systematis nervosa centralis  Tuberculosis meningum alia definite  Meningitis miliaris tuberculosa  Tuberculoma systematis nervosi centralis  Tuberculoma systematis nervosi centralis alia  Tuberculosis of nervous system (all subgroups) |
| Other  ICD 8*  ICD 10* | 4699  94  DA521  DA522  DA523  DA81 | Enterovirus systematis nervosa centralis alius  Syphilis of central nervous system (all subgroups)  Symptomatic neurosyphilis  Asymptomatic neurosyphilis  Neurosyphilis, unspecified  Atypical virus infections of central nervous system (all subgroups except DA811 and DA812 categorized as encephalitis) |

* Diagnoses codes according to the International Classification of Diseases (ICD) 8th revision until December 1993 and 10th revision thereafter.
